# Antifungal and anti-biofilm effects of hydrazone derivatives on *Candida* spp

**DOI:** 10.1080/14756366.2024.2429109

**Published:** 2024-11-26

**Authors:** Pierre Popczyk, Alina Ghinet, Clovis Bortolus, Laure Kamus, Marc F. Lensink, Jérôme de Ruyck, Boualem Sendid, Faustine Dubar

**Affiliations:** a INSERM U1285, Université de Lille, CHU de Lille, UMR CNRS 8576-UGSF-Unité de Glycobiologie Structurale et Fonctionnelle, Lille, France; b Junia, Health and Environment, Laboratory of Sustainable Chemistry and Health, Lille, France; c UMR 1167 – RID-AGE – Risk Factors and Molecular Determinants of Aging-Related Diseases, Univ. Lille, Inserm, CHU Lille, Institut Pasteur de Lille, Lille, France; d Alexandru Ioan Cuza University of Iasi, Iasi, Romania; e Department of Medical Biology, Félix-Guyon Hospital Center, Saint-Denis, France; f UMR Processus Infectieux en Milieu Insulaire Tropical (PIMIT), CNRS 9192, INSERM U1187, IRD 249, Université de La Réunion, Saint-Denis, France; g Univ. Lille, CNRS, UMR 8576-UGSF-Unité de Glycobiologie Structurale et Fonctionnelle, Lille, France

**Keywords:** Antifungal, hydrazone, trehalose, *Galleria mellonella*, biofilm, *Candida*

## Abstract

Worldwide, invasive candidiasis are a burden for the health system due to difficulties to manage patients, to the increasing of the resistance of the current therapeutics and the emergence of naturally resistant species of *Candida*. In this context, the development of innovative antifungal drugs is urgently needed. During invasive candidiasis, yeast is submitted to many stresses (oxidative, thermic, osmotic) in the human host. In order to resist in this context, yeast develops different strategy, especially the biosynthesis of trehalose. Starting from the 3D structural data of TPS2, an enzyme implicated in trehalose biosynthesis, we identified hydrazone as an interesting scaffold to design new antifungal drugs. Interestingly, our hydrazone derivatives which demonstrate antifungal and anti-biofilm effects on *Candida spp*., are non-toxic in *in vitro* and *in vivo* models (*Galleria mellonella*).

## Introduction

Invasive fungal infections (IFIs) contribute to significant morbidity and mortality worldwide^1^. Each year, these diseases are estimated to result in 300 million cases of severe fungal infection and approximately 1.5 million deaths worldwide^2^. Alarmingly, the incidence of these infections is increasing across the world^3^. In France, the French National Reference Centre for Invasive Mycoses and Antifungals records an increasing of the incidence of fungemia between 2012 and 2018 (1.03–1.19/10,000, *p* = .0023)^4^. Among the opportunistic fungi, *Candida* is an aggressive pathogen that is normally held in check by the immune system in healthy people. However, people with a weakened immune system, including those with HIV, autoimmune diseases, organ transplantations or cancers, are susceptible to *Candida* infections, which can be life-threatening. Furthermore, advances in medical management such as antineoplastic chemotherapy, organ transplantation, haemodialysis, parenteral nutrition, and central venous catheters also contribute to fungal invasion and colonisation. Pathogenic fungal species generate exorbitant costs for the healthcare system due to the complexity of the management of infected patients^1^^,^^5^. These difficulties result from a lack of specific and sensitive diagnostic tools, a limited therapeutic arsenal, but also an increase in antifungal resistance or the emergence of strains that are naturally resistant to current antifungals. Despite a decline in infections caused by *Candida albicans*, the most common *Candida* spp.^6^, there has been an increase in infections caused by other *Candida* spp^7^^,^^8^. The increase in infections caused by *Candida kefyr*, *Clavispora lusitaniae, Candida haemulonii*^9^ or *Candida auris*, which are species that are frequently resistant to current antifungal drugs, are causing changes in treatment guidelines^10^.

The antifungal therapeutic arsenal includes four broad categories of drugs: polyenes, azoles, echinocandins, and pyrimidine analogues^11^. Despite the therapeutic success of these drugs, there has been an acceleration in drug resistance to these antifungal classes^12^. In addition, the relevance of topics related to the resistance of infectious diseases (a major axis of the World Health Organisation) was mentioned in the final report of the G20 summit in 2017–2018 as a public health priority^13^. Thus, a current major challenge is to identify new therapeutic targets.

Since IFIs concern immunocompromised patients, the therapeutic/toxicity ratio must also be highly favourable. Innovative targets should be essential pathways of fungal metabolism to design molecules that are not toxic to the host. In order to successfully colonise the host, *Candida* spp. need to use reserve carbohydrates for energy^14^ and require protection against the host’s immune system, which puts yeast cells under significant stress^15^. Among the reserve carbohydrates, trehalose, a non-reductive disaccharide with two glucose units bound by an α-1,1 glycosidic bond, is described as an alternative carbon source for some fungi^16^. The role of trehalose biosynthesis is central to a wide variety of organisms, including fungal pathogens as *Candida*. Protective mechanisms against different stresses have been proposed to be mediated by the disaccharide trehalose^17^^,^^18^. Genes coding for proteins involved in trehalose biosynthesis are related to fungal metabolism, cell wall homeostasis, stress responses, and virulence^19^. Furthermore, trehalose has also been shown to be absent from mammalian cells under physiological conditions^20^. As a result, the biosynthetic pathways leading to the formation of this disaccharide and their use as potential targets for the development of new antifungal agents appear attractive^21^.

**Figure 1.**
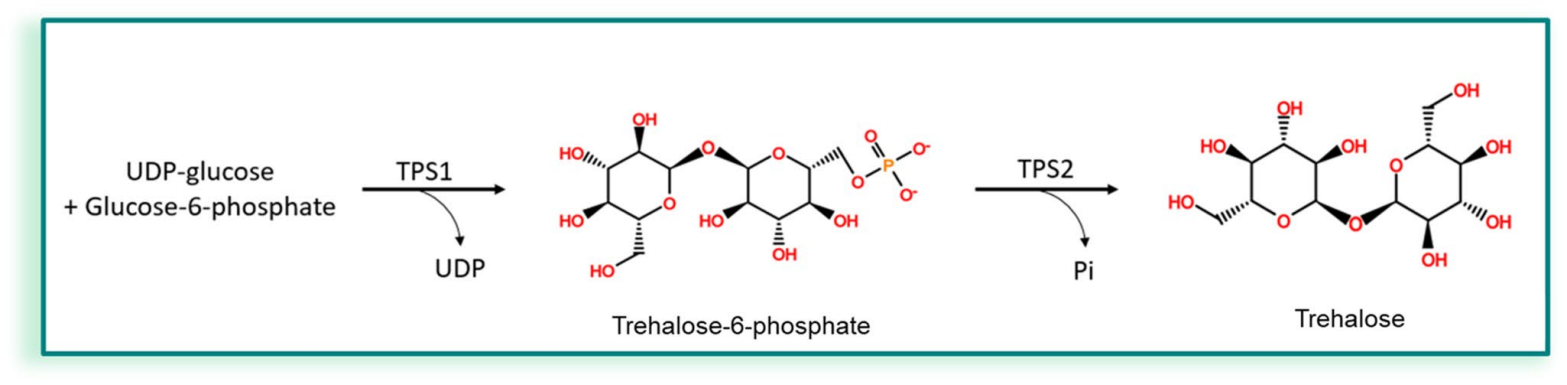
Canonical pathway for the synthesis of trehalose in *Candida.*

In *Candida* spp., the TPS1/TPS2 biosynthetic pathway is considered as the canonical pathway^22^ (Figure 1) and involves two main enzymes: trehalose-6-phosphate synthase (TPS1) and trehalose-6-phosphate phosphatase (TPS2). TPS1 converts UDP-glucose and glucose-6-phosphate to UDP and trehalose-6-phosphate (T6P). T6P is then converted to trehalose by TPS2. Interestingly, structural data for the enzymes TPS1 and TPS2 are available.

In many yeast species, a deficiency in TPS1 results in increased sensitivity to stress conditions, an alteration in biofilm development, and lower infection rates in mice^23^^,^^24^. In addition, strains deficient in TPS2 are more sensitive to oxidation, have a lower resistance to destruction by phagocytes, and have reduced virulence^23–27^. The absence of TPS2 causes the build-up of intermediate T6P, which is toxic to yeasts. Moreover, deletion of TPS2 results in strongly reduced virulence of *C. albicans* due to the accumulation of T6P instead of trehalose in response to stress. Moreover, the TPS2Δ strain is also thermosensitive and its cell integrity pathways are affected. Overall, it was shown that deletion of TPS2 had an even stronger effect than deletion of TPS1.

Some studies have investigated the development of TPS1 inhibitors^28^^,^^29^. A recent study showed that T6P inhibits TPS1 activity *in vitro* and represents a promising candidate for the rational design of antifungal drugs^30^. Concerning TPS2, two studies report the design, synthesis, and evaluation of a library of aryl D-glucopyranoside-6-sulphates as reversible inhibitors of TPS2. The evaluation of inhibitory activity was carried out using different TPS2 orthologues derived from parasites, mycobacteria, and bacteria, but not fungi. These new reversible inhibitors do not have chemical structures close to those of current antifungal drugs and the chemical space covered by these inhibitors is not very wide since they are T6P-analogues.

In many yeast species, TPS2-deficient strains are more susceptible to oxidation, have lower resistance to phagocytosis and have reduced virulence^23–27^. Interestingly, structural data on TPS2 are available^31^ and can be used as a guide for the design of therapeutically oriented molecules specifically targeting these enzymes of interest.

High throughput screening (HTS) is commonly used for novel drug discovery. Among the different available HTS techniques, computational *de novo* drug design^32^ represents a promising tool to produce novel molecular entities with the required pharmacological profiles. These design methods aim to create new molecules with steric and electrostatic compatibility for the target system. Conceptually, the approach has some parallels with the design of fragment-based drugs. The advantage of this method is that it is not limited to existing databases and makes it possible to explore many regions of chemical space.

During this project, we carried out a *de novo design* study of the TPS2 enzyme structure to design TPS2-inhibitor scaffolds. The *de novo design* study allowed the identification of potential antifungal compounds. Almost 500 compounds were screened (unpublished results) and the hydrazone moiety was identified as a promising scaffold to develop new antifungal drugs. The present study evaluates the toxicity and antifungal effect of hydrazones derivatives.

## Methods

Starting materials were purchased from TCI chemicals and Merck, and were used without purification. Nuclear magnetic resonance (NMR) spectra were acquired at room temperature, at 300 MHz for ^1^H-NMR, 75.4 MHz for ^13^C-NMR, and 282 MHz for ^19^F-NMR on a Bruker AC300 spectrometer (University of Lille, France) with tetramethylsilane (TMS) as an internal standard. Chemical shifts (δ) are expressed in ppm relative to TMS. Splitting patterns are designated as: s, singlet; d, doublet; dd, doublet of doublets; ddd, doublet of doublet of doublets; t, triplet. Coupling constants (*J*) are reported in Hertz (Hz). Thin layer chromatography (TLC) was carried out on Macherey Nagel silica gel plates (Macherey Nagel, Hoerdt, France) with a fluorescent indicator and were visualised under a UV-lamp at 254 nm and 365 nm. Elemental analyses (C, H, N) of synthesized compounds were determined on a Thermo Scientific^TM^ FLASH^TM^ 2000 Organic Elemental Analyser (Service d’Analyses, de Mesures Physiques et de Spectroscopie Optique, Fédération de Chimie Le Bel, Strasbourg, France).

### Computational study

The starting structure for all modelling was the crystal structure of the C-terminal domain of *C. albicans* T6P phosphatase^31^ (PDB ID 5DXI, 2 Å). Virtual screening of the ZINC database was performed using GOLD^33^ based on a genetic algorithm with optimised parameters for high-throughput virtual screening.

The *de novo* design was performed using LigBuilder 3.0^34^. LigBuilder is based on molecular mechanics and has two main functional modules (Cavity and Build). The cavity is designed to analyse the binding pocket of the target and to prepare the data necessary to run Build. Build is the main functional module for *de novo* design and results analysis. Subsequent docking simulations were performed using GOLD (See supplemental information for a sequence in FASTA format). The docking search was centered on the detected cavity (I33, H87H139) with a radius of 15 Å covering a large part of the domain. Default settings were used for the search and the scoring function was ChemPLP.

All the pictures for the computational procedure were generated using PyMOL^35^.

### General procedure for the synthesis of hydrazones

Glacial acetic acid (225 µL) was added dropwise to a suspension of benzaldehyde derivative (0.9 mmol, 1 equiv) and hydrazine derivative (0.9 mmol, 1 equiv) in absolute ethanol (1.6 ml). The mixture was stirred at reflux for 12 h. After this time, the mixture was cooled at room temperature and then dissolved in 20 mL of ethyl acetate. The organic layer was washed successively with 20 mL of NaOH aqueous solution (0.1 N) and then 20 mL of distilled water. After drying on anhydrous MgSO_4_ and filtration, the organic layers were evaporated under reduced pressure.

(*E*)-1–(4-bromophenyl)-2–(2-nitrobenzylidene)hydrazine (**PP02**)^36^


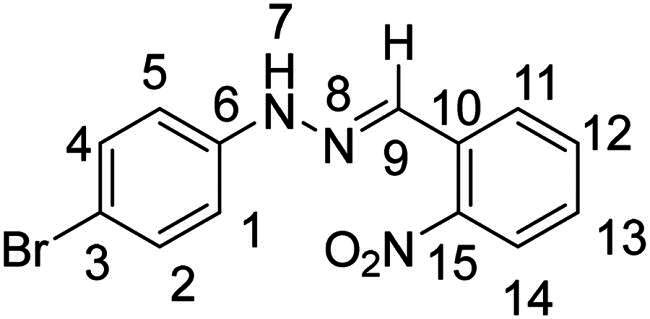


The general procedure was used with 2-nitrobenzaldehyde (0.135 g) and 4-bromophenylhydrazine hydrochloride (0.166 g) to obtain the pure product **PP02** as a red solid (0.286 g, yield = quant). m.p. 175 °C–177 °C.

^1^H NMR (300 MHz, DMSO-*d_6_*) δ 11.00 (s, H7), 8.26 (s, H9), 8.15 (dd, *J* = 8.0, 1.4 Hz, H11), 7.98 (dd, *J* = 8.2, 1.3 Hz, H14), 7.71 (m, H12), 7.51 (ddd, *J* = 8.6, 7.3, 1.4 Hz, H13), 7.45 − 7.36 (m, H2 and H4), 7.10–7.01 (m, H1 and H5).^13^C NMR (75.4 MHz, DMSO-*d_6_*) δ 147.4 (C15), 144.4 (C6), 133.7 (C13), 132.4 (C2 and C4), 132.3 (C9), 130.1 (C10), 128.9 (C12), 127.6 (C11), 125.1 (C14), 114.8 (C1 and C5), 111.2 (C3). Elemental analysis calcd (%) for C_13_H_10_BrN_3_O_2_: C 48.77, H 3.15, Br 24.96, N 13.13, O 10.00; found C 48.96, H 3.18, N 13.12.

(*E*)-2–(2-benzylidenehydrazinyl)-5-bromopyridine (**PP04**)^37^


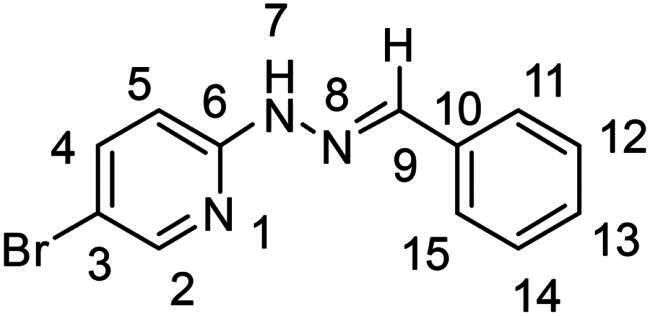


The general procedure was used with benzaldehyde (0.094 g) and 5-bromo-2-hydrazinopyridine (0.167 g) to obtain the pure product **PP04** as a white solid (0.281 g, yield= 96%). m.p. 205 °C–207 °C.

^1^H NMR (300 MHz, DMSO-*d_6_*) δ 11.08 (s, H7), 8.19 (sd, *J* = 2.5, 0.6 Hz, H2), 8.03 (s, H9), 7.81 (dd, *J* = 8.9, 2.4 Hz, H4), 7.71–7.61 (m, H11 and H15), 7.46–7.28 (m, H12, H13 and H14), 7.22 (dd, *J* = 8.9, 0.7 Hz, H5).^13^C NMR (75.4 MHz, DMSO-*d_6_*) δ 156.4 (C6), 148.6 (C2), 140d$.7 (C4), 140.3 (C9), 135.6 (C10), 129.2 (C12, C13 and C14), 126.6 (C11 and C15), 108.8 (C5), 108.8 (C3). Elemental analysis calcd (%) for C_12_H_10_BrN_3_: C 52.20, H 3.65, Br 28.94, N 15.22; found C 50.25, H 3.63, N 14.2.

(*E*)-1–(3,5-dichlorophenyl)-2–(2-nitrobenzylidene)hydrazine (**PP11**)


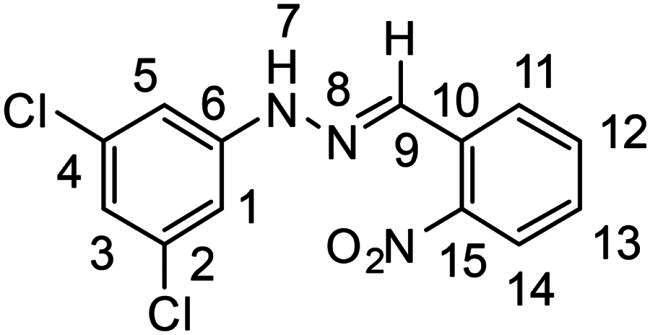


The general procedure was used with 2-nitrobenzaldehyde (0.142 g) and 3,5-dichlorophenylhydrazine hydrochloride (0.200 g) to obtain the pure product **PP11** as a brown/red solid (0.200 g, yield= 69%). m.p. 216 °C.

^1^H NMR (300 MHz, DMSO-*d_6_*) δ 11.17 (s, H7), 8.28 (s, H9), 8.18 (dd, *J* = 8.0, 1.4 Hz, H11), 7.99 (dd, *J* = 8.2, 1.3 Hz, H14), 7.74 (m, H12), 7.55 (ddd, *J* = 8.2, 7.3, 1.4 Hz, H13), 7.04 (d, *J* = 1.9 Hz, H1 and H5), 6.93 (t, *J* = 1.9 Hz, H3). ^13^C NMR (75.4 MHz, DMSO-*d_6_*) δ 147.7 (C15), 147.4 (C6), 135.3 (C10), 134.6 (C9), 133.8 (C12), 129.6 (C2 and C4), 129.5 (C13), 128.1 (C11), 125.0 (C14), 118.8 (C3), 111.1 (C1 and C5). Elemental analysis calcd (%) for C_13_H_9_Cl_2_N_3_O_2_: C 50.35, H 2.92, Cl 22.86, N 13.55, O 10.32; found C 42.56, H 2.42, N 11.53.

(*E*)-1–(2,4-difluorophenyl)-2–(2-nitrobenzylidene)hydrazine (**PP12**)


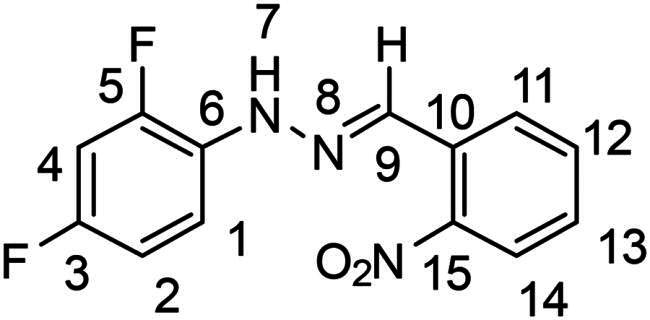


The general procedure was used with 2-nitrobenzaldehyde (0.167 g) and 2,4-difluorophenylhydrazine hydrochloride (0.200 g) to obtain the pure product **PP12** as an orange solid (0.121 g, yield= 40%). m.p. 167 °C.

^1^H NMR (300 MHz, DMSO-*d_6_*) δ 10.78 (s, H7), 8.48 (s, H9), 8.14 (dd, *J* = 8.0, 1.4 Hz, H14), 7.97 (dd, *J* = 8.2, 1.3 Hz, H11), 7.71 (m, H12), 7.58–7.43 (m, H1 and H13), 7.25 (ddd, *J* = 11.8, 8.9, 2.8 Hz, H4), 7.02 (m, H2). ^13^C NMR (75.4 MHz, DMSO-*d_6_*) δ 157.3–154.2 (dd, *J* = 234.7, 10.4 Hz, C5), 150.7–147.5 (dd, *J* = 239.9, 11,9 Hz, C3), 147.6 (C15), 134.0 (C9), 133.6 (C12), 130.2 (dd, *J* = 9.5, 3.0 Hz, C6), 129.9 (C10), 129.2 (C13), 127.7 (C11), 124.9 (C14), 115 (dd, *J* = 8.9, 8.9 Hz, C1), 112.1 (dd, *J* = 21.6, 3 Hz, C2), 104.5 (dd, *J* = 26.8, 26.8 Hz, C4). ^19^F NMR (282 MHz, DMSO-*d_6_*) δ −122.6, −129,1. Elemental analysis calcd (%) for C_13_H_9_F_2_N_3_O_2_: C 56.63, H 3.27, F 13.71, N 15.16, O 11.54; found C 55.95, H 3.31, N 14.91.

(*E*)-*N’*-(2-nitrobenzylidene)-[1,1′-biphenyl]-4-carbohydrazide (**PP13**)


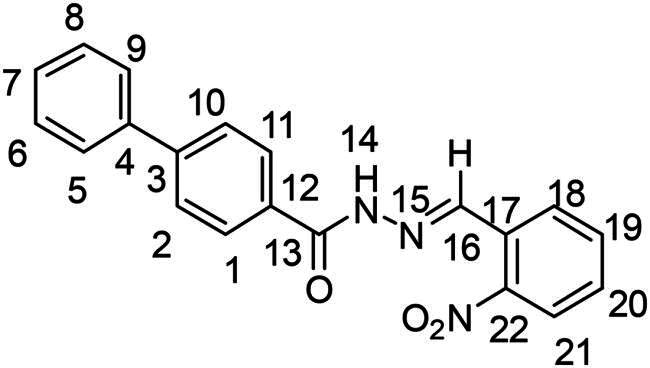


The general procedure was used with 2-nitrobenzaldehyde (0.140 g) and biphenyl-4-carboxylic hydrazide (0.200 g) to obtain the pure product **PP13** as a white solid (0.108 g, yield= 33%). m.p. 203 °C–204 °C.

^1^H NMR (300 MHz, DMSO-*d_6_*) δ 12.28 (s, H14), 8.91 (s, H16), 8.16 (d, *J* = 7.7 Hz, H18), 8.13–8.01 (m, H2, H10 and H21), 7.85 (m, H5-H9-H19), 7.80–7.74 (m, H1 and H11), 7.69 (m, H20), 7.51 (m, H6 and H8), 7.47–7.40 (m, H7). ^13^C NMR (75.4 MHz, CDCl_3_) δ 163.4 (C13), 148.7 (C22), 144.0 (C3), 143.3 (C16), 139.5 (C4), 134.2 (C19), 132.2 (C12), 131.1 (C20), 129.5 (C6 and C8), 129.2 (C17), 128.9 (C2 and C10), 128.7 (C7), 128.4 (C18), 127.4 (C1 and C11), 127.2 (C5 and C9), 125.1 (C21). Elemental analysis calcd (%) for C_20_H_15_N_3_O_3_: C 69.56, H 4.38, N 12.17, O 13.90; found C 69.69, H 4.45, N 12.03.

(*E*)-1–(2-ethylphenyl)-2–(2-nitrobenzylidene)hydrazine (**PP14**)


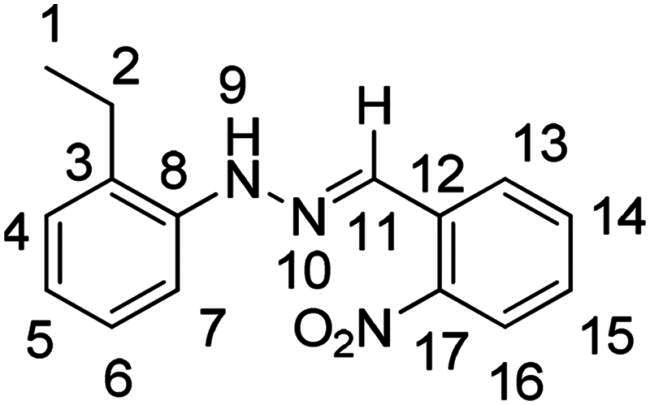


The general procedure was used with 2-nitrobenzaldehyde (0.175 g) and 2-ethylphenylhydrazine hydrochloride (0.200 g) to obtain the pure product **PP14** as a red solid (0.142 g, yield= 45%). m.p. 87 °C–88 °C.

^1^H NMR (300 MHz, DMSO-*d_6_*) δ 10.23 (s, H9), 8.56 (s, H11), 8.18 (dd, *J* = 8.0, 1.4 Hz, H16), 7.95 (dd, *J* = 8.2, 1.2 Hz, H13), 7.71 (m, H14), 7.55–7.39 (m, H7 and H15), 7.12 (m, H4 and H6), 6.82 (td, *J* = 7.4, 1.3 Hz, H5), 2.63 (q, *J* = 7.5 Hz, H2), 1.16 (t, *J* = 13.0, 5.5 Hz, H1). ^13^C NMR (75.4 MHz, DMSO-*d_6_*) δ 147.5 (C17), 142.3 (C8), 133.5 (C14), 132.1 (C11), 130.4 (C12), 129.2 (C6), 128.7 (C15), 127.7 (C3), 127.3 (C4), 127.2 (C13), 124.9 (C16), 120.6 (C5), 113.4 (C7), 23.7 (C2), 14.7 (C1). Elemental analysis calcd (%) for C_15_H_15_N_3_O_2_: C 66.90, H 5.61, N 15.60, O 11.88; found C 67.07, H 5.62, N 14.56.

2-(phenylmethylene)hydrazinecarboximidamide (**PP16**)


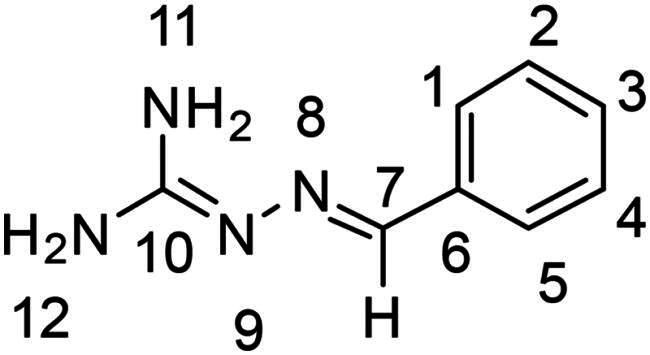


The general procedure was used with benzaldehyde (0.192 g) and aminoguanidine hydrochloride (0.200 g) to obtain the pure product **PP16** as a white solid (0.133 g, yield= 45%). m.p. 176 °C.

^1^H NMR (300 MHz, DMSO-*d_6_*) δ 7.99 (s, H7), 7.74–7.58 (d, H1 and H5), 7.42–7.17 (m, H2, H3 and H4), 5.90 (s, H12), 5.52 (s, H11). ^13^C NMR (75.4 MHz, DMSO-*d_6_*) δ 161.1 (C10), 143.6 (C7), 137.5 (C6), 128.8 (C2 and C4), 128.2 (C3), 126.7 (C1 and C5). Elemental analysis calcd (%) for C_8_H_10_N_4_: C 59.24, H 6.21, N 34.54; found C 58.12, H 6.20, N 33.05.

(*E*)-1-benzylidene-2–(4-bromophenyl)hydrazine (**PP17**)^38^


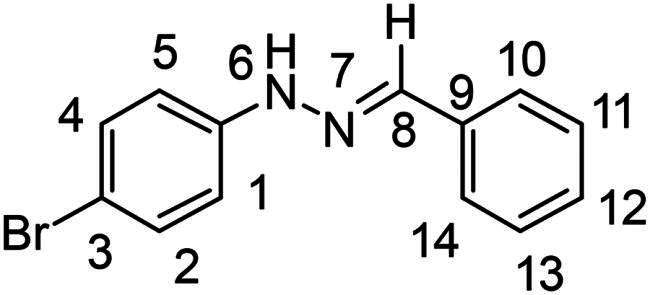


The general procedure was used with benzaldehyde (0.094 g) and 4-bromophenylhydrazine hydrochloride (0.166 g) to obtain the pure product **PP17** as a pink solid (0.245 g, yield = quant). m.p. 125 °C–126 °C.

^1^H NMR (300 MHz, DMSO-*d_6_*) δ 10.49 (s, H6), 7.88 (s, H8), 7.71–7.60 (m, H2-H4), 7.48–7.27 (m, H10, H11, H12, H13 and H14), 7.11–6.98 (m, H1 and H5). ^13^C NMR (75.4 MHz, DMSO-*d_6_*) δ 145.1 (C6), 137.9 (C8), 136.0 (C9), 132.2–128.9 (C10, C11, C12, C13 and C14), 126.3 (C2 and C4), 114.4 (C1 and C5), 110.0 (C3). Elemental analysis calcd (%) for C_13_H_11_BrN_2_: C56.75, H 4.03, Br 29.04, N 10.18; found C 56.67, H 3.96, N 10.24.

2-[(2-nitrophenyl)methylene]hydrazinecarboximidamide (**PP25**)


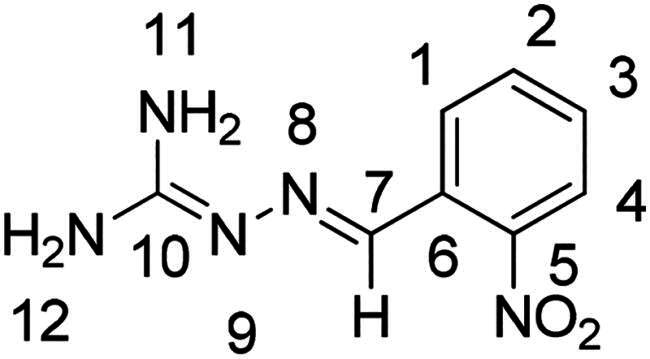


The general procedure was used with 2-nitrobenzaldehyde (0.273 g) and aminoguanidine hydrochloride (0.200 g) to obtain the pure product **PP25** as an orange solid (0.148 g, yield= 40%). m.p. 177 °C.

^1^H NMR (300 MHz, DMSO-*d_6_*) δ 8.27 (dd, *J* = 8.0, 1.4 Hz, H1), 8.21 (s, H7), 7.87 (dd, *J* = 8.2, 1.3 Hz, H4), 7.61 (ddd, *J* = 8.0, 7.3, 1.3 Hz, H2), 7.43 (ddd, *J* = 8.1, 7.3, 1.5 Hz, H3), 6.08 (s, H12), 5.81 (s, H11). ^13^C NMR (75.4 MHz, DMSO-*d_6_*) δ 162.2 (C10), 147.7 (C5), 137.2 (C7), 133.0 (C2), 131.2 (C6), 128.3 (C3) 128.2 (C1), 124.6 (C4). Elemental analysis calcd (%) for C_8_H_9_N_5_O_2_: C 46.38, H 4.38, N 33.80, O 15.44; found C 46.66, H 4.63, N 32.37.

(*E*)-5-bromo-2–(2-(2-nitrobenzylidene)hydrazinyl)pyridine (**PP28**)


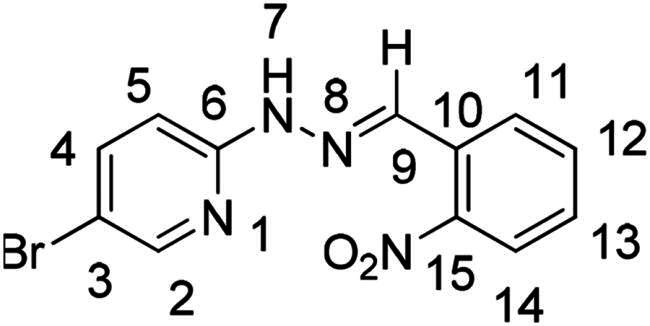


The general procedure was used with 2-nitrobenzaldehyde (0.160 g) and 5-bromo-2-hydrazinopyridine (0.200 g) to obtain the pure product **PP28** as an orange solid (0.278 g, yield= 81%). m.p. 211 °C–212 °C.

^1^H NMR (300 MHz, DMSO-*d_6_*) δ 11.46 (s, H7), 8.40 (s, H9), 8.23 (dd, *J* = 2.5, 0.7 Hz, H2), 8.14 (dd, *J* = 8.0, 1.4 Hz, H11), 7.98 (dd, *J* = 8.2, 1.1 Hz, H14), 7.85 (dd, *J* = 8.9, 2.5 Hz, H4), 7.73 (tdd, *J* = 8.0, 1.4, 0.6 Hz, H12), 7.55 (ddd, *J* = 8.2, 7.4, 1.4 Hz, H13), 7.23 (dd, *J* = 8.9, 0.7 Hz, H5). ^13^C NMR (75.4 MHz, DMSO-*d_6_*) δ 155.9 (C6), 148.7 (C2), 147.8 (C15), 140.9 (C4), 134.8 (C9), 133.7 (C12), 129.7 (C13), 129.5 (C10), 127.9 (C11), 124.9 (C14), 109.9 (C3), 109.1 (C5). Elemental analysis calcd (%) for C_12_H_9_BrN_4_O_2_: C 44.88, H 2.82, Br 24.88, N 17.45, O 9.96; found C 45.35, H 2.89, N 17.51.

(*E*)-1–(3,4-dimethylphenyl)-2–(2-nitrobenzylidene)hydrazine (**PP29**)


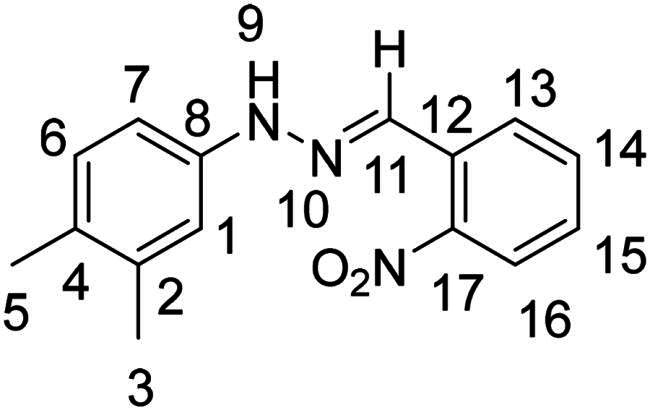


The general procedure was used with 2-nitrobenzaldehyde (0.175 g) and 3,4-dimethylphenylhydrazine hydrochloride (0.200 g) to obtain the pure product **PP29** as a purple red solid (0.107 g, yield= 34%). m.p. 137 °C–138 °C.

^1^H NMR (300 MHz, DMSO-*d_6_*) δ 10.75 (s, H9), 8.22 (s, H11), 8.16 (dd, *J* = 8.0, 1.4 Hz, H13), 7.95 (dd, *J* = 8.2, 1.1 Hz, H16), 7.68 (m H14), 7.45 (ddd, *J* = 8.2, 7.3, 1.5 Hz, H15), 7.00 (d, *J* = 8.2 Hz, H6), 6.92 (d, *J* = 2.0 Hz, H1), 6.83 (dd, *J* = 8.1, 2.4 Hz, H7), 2.19 (s, H3), 2.13 (s, H5). ^13^C NMR (75.4 MHz, DMSO-*d_6_*) δ 147.0 (C17), 143.0 (C8), 137.4 (C2), 133.6 (C14), 130,7 (C12), 130.6 (C6), 130.1 (C11), 128.3 (C9), 127.9 (C4), 127.3 (C13), 125.0 (C16), 114.2 (C1), 110.5 (C7), 20.3 (C3), 19.1 (C5). Elemental analysis calcd (%) for C_15_H_15_N_3_O_2_: C 66.90, H 5.61, N 15.60, O 11.88; found C 66.91, H 5.56, N 15.61.

(*E*)-1-benzylidene-2–(3,5-dichlorophenyl)hydrazine (**PP49**)^39^


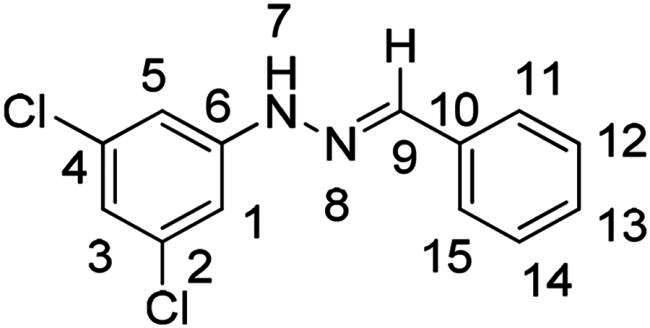


The general procedure was used with benzaldehyde (0.099 g) and 3,5-dichlorophenylhydrazine hydrochloride (0.200 g) to obtain the pure product **PP49** as a red solid (0.177 g, yield= 71%). m.p. 111 °C–114 °C.

^1^H NMR (300 MHz, DMSO-*d_6_*) δ 10.71 (s, H7), 7.91 (s, H9), 7.73–7.65 (m, H1 and H5), 7.45–7.28 (m, H3, H12 and H14), 7.02 (d, *J* = 1.9 Hz, H11 and H15), 6.84 (t, *J* = 1.9 Hz, H13). ^13^C NMR (75.4 MHz, DMSO-*d_6_*) δ 148.0 (C6), 139.9 (C9), 135.5 (C10), 135.2 (C2 and C4), 129.2 (C1 and C5), 126.6 (C3, C12 and C14), 117.8 (C13), 110.6 (C11 and C15). Elemental analysis calcd (%) for C_13_H_10_Cl_2_N_2_: C 58.89, H 3.80, Cl 26.74, N 10.57; found C 59.32, H 3.86, N 10.16.

(*E*)-1-benzylidene-2–(2,4-difluorophenyl)hydrazine (**PP50**)^40^


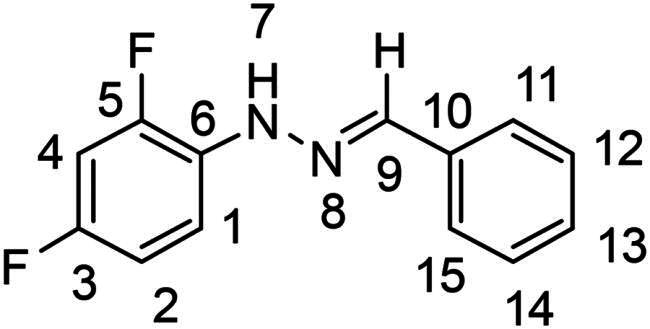


The general procedure was used with benzaldehyde (0.117 g) and 2,4-difluorophenylhydrazine hydrochloride (0.200 g) to obtain the pure product **PP50** as a red solid (0.235 g, yield= 91%). m.p. 81 °C.

^1^H NMR (300 MHz, DMSO-*d_6_*) δ 10.21 (s, H7), 8.12 (s, H9), 7.72–7.58 (m, H11 and H15), 7.50 (td, *J* = 9.3, 5.9 Hz, H1), 7.44–7.35 (m, H12 and H14), 7.35–7.26 (m, H13), 7.20 (ddd, *J* = 11.9, 8.9, 2.8 Hz, H2), 7.00 (tdd, *J* = 8.6, 2.9, 1.5 Hz, H4). ^13^C NMR (75.4 MHz, DMSO-*d_6_*) δ 156.8–153.6 (dd, *J* = 233.9, 10.4 Hz, C5) 150.5–147.3 (dd, *J* = 239.1, 11.9 Hz, C3), 139.9 (C9), 136.0 (C10), 131,0 (dd, *J* = 10.4, 3.0 Hz, C6), 129.1 (d, *J* = 23.8 Hz, C6), 129.2 (C12 and C14), 128.9 (C13), 126.4 (C11 and C15), 114.6 (dd, *J* = 8.9, 8.9 Hz, C1), 112.0 (dd, *J* = 21.6, 3 Hz, C4), 104.3 (dd, *J* = 26.8, 26.1 Hz, C2). ^19^F NMR (282 MHz, DMSO-*d_6_*) δ −124.1, −129.7. Elemental analysis calcd (%) for C_13_H_10_F_2_N_2_: C 67.24, H 4.34, F 16.36, N 12.06; found C 66.77, H 4.26, N 9.90.

(*E*)-1-(*tert*-butyl)-2–(2-nitrobenzylidene)hydrazine (**PP53**)^41^


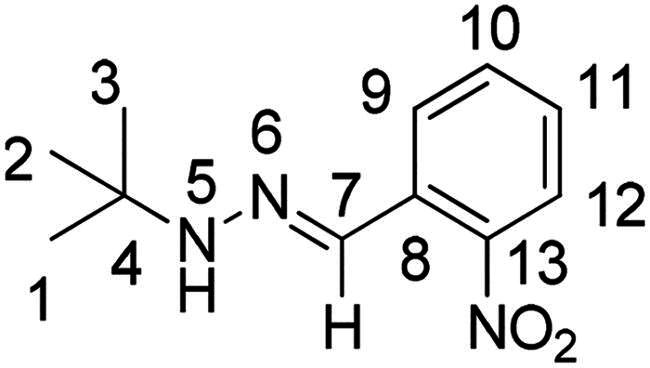


The general procedure was used with 2-nitrobenzaldehyde (0.242 g) and *tert*-butylhydrazine hydrochloride (0.200 g) to obtain the pure product **PP53** as a red solid (0.291 g, yield= 82%). m.p. 66 °C.

^1^H NMR (300 MHz, DMSO-*d_6_*) δ 7.95 (m, H7 and H9), 7.83 (m, H5 and H12), 7.60 (m, H10), 7.36 (ddd, *J* = 8.1, 7.3, 1.4 Hz, H11), 1.16 (s, H1, H2 and H3). ^13^C NMR (75.4 MHz, DMSO-*d_6_)* δ 146.7 (C13), 133.1 (C10), 131.1 (C8), 128.2 (C7), 127.5 (C11), 126.9 (C9), 124.7 (C12), 53.9 (C4), 29.0 (C1, C2 and C3). Elemental analysis calcd (%) for C_11_H_15_N_3_O_2_: C 59.71, H 6.83, N 18.99, O 14.46; found C 60.19, H 6.70, N 18.58.

### Hydrazone solution preparation

Hydrazones (2–5 mg) were dissolved in 1 mL DMSO to obtain a stock solution. The stock solution was then dissolved in mQ water in order to obtain a standard solution of hydrazone at a final concentration of 32 µg/mL. This standard solution was used for the successive dilution method to obtain a concentration range of molecules from 0.25–32 µg/mL.

### Fungal culture

The *Candida* strains (Table 1) were cultured on Sabouraud agar medium for 12 h and then in liquid Sabouraud medium (Sigma S3306) on a rotary shaker for 18 h at 37 °C. The fungal cultures obtained were then centrifuged at 600 rpm for 3 min and washed in phosphate-buffered saline (PBS; Gibco; 14200–067).

**Table 1.** *Candida* strains, origin, reference and resistance profile.

Strains	*C. albicans*	*C. albicans*	*C. albicans*	*C. glabrata*	*C. glabrata*
Origin and reference	ATCC SC5314	CHU 17287C305	CHU 14402C5521	ATCC 2001	CHU 16374C3785
Resistance	No	Echinocandins	Azoles	No	Azoles Echinocandins
Strains	*C. auris*	*C. auris*	*C. dubliniensis*	*C. tropicalis*	*C. parapsilosis*
Origin and reference	CHU 1.4.4.1.69	CHU 1.4.4.12.29	CHU 22114C2813	CHU 17211M323	CHU 14482C4760
Resistance	Azoles Echinocandins	No	No	Azoles	No

### Human embryonic kidney 293 cell line

HEK-293 cells are derived from a human embryonic kidney cell line (ATCC CRL-1573). The culture was carried out in Dulbecco’s Modified Eagle’s Medium (DMEM; Gibco 61965–026) supplemented with 10% foetal calf serum and 1% antibiotic (penicillin-streptomycin; 15140–122 100 ml; Gibco). The flask containing the cell culture was placed in an incubator at 37 °C containing 5% CO_2_.

### Primary screening

Primary screening assays against *Candida* spp. were performed according to the standard culture microdilution method from the Clinical and Laboratory Standard Institute (CLSI). Inocula from *Candida* spp. strains were obtained from fungal cultures on Sabouraud dextrose agar (yeast extract 0.5%, tryptone 1%, NaCl 1%, agar 1.2% and amikacin) at 37 °C for 24 h. The initial concentration of *Candida* strains was 1–5 × 10^6^ CFU/mL. The inocula were adjusted in order to obtain an optical density of 0.5 on the McFarland scale using PBS. Yeasts were suspended in RPMI 1640 medium (Roswell Park Memorial Institute medium) to obtain a final concentration of 5 × 10^2^ CFU/mL per well. Evaluation of the antifungal activity of hydrazone derivatives was performed against *C. albicans* SC5314, *C. albicans* clinical strain, *C. albicans* R-echinocandins, *C. auris*, *C. auris* clinical strain, *C. dubliniensis*, *C. tropicalis*, *C. parapsilosis*, C*. glabrata* R-azoles and *C. glabrata* R-echinocandins cultured in 96-well microplates at a single concentration of 32 μg/mL at 37 °C for 24 h. Growth and sterility controls were also used. A positive control was also included with DMSO 10% and a negative control with DMSO 1%. Fungal growth was determined in colorimetric assays by direct visualisation (Alamarblue^TM^, Biorad, France). The yeasts were considered dead when the colorimetric stain stayed blue.

### MIC_99_ assays

MIC_99_ determination against *Candida* spp. was performed according to the standard culture microdilution method from the CLSI. *Candida* spp. strains were obtained from fungal cultures on Sabouraud dextrose agar at 37 °C for 24 h. The initial concentration of *Candida* strains was 1–5 × 10^6^ CFU/mL. The inocula were adjusted in order to obtain an optical density of 0.5 on the McFarland scale using PBS. Yeasts were suspended in RPMI 1640 medium (Roswell Park Memorial Institute medium) to obtain a final concentration of 5 × 10^2^ CFU/mL per well. Evaluation of the antifungal activity of hydrazone derivatives was performed against *C. albicans* SC5314, *C. albicans* clinical strain, *C. albicans* R-echinocandins, *C. auris*, *C. auris* clinical strain, *C. dubliniensis*, *C. tropicalis*, *C. parapsilosis*, C*. glabrata* R-azoles and *C. glabrata* R-echinocandins cultured in 96-well microplates at different concentrations (0.25 μg/mL–32 μg/mL) at 37 °C for 24 h. Growth and sterility controls were also used. A positive control was also included with DMSO 10% and a negative control with DMSO 1%. Fungal growth was determined in colorimetric assays (Alamarblue^TM^, Biorad, France). MIC was defined as the lowest concentration of hydrazone derivative that allows a reduction of 99% of yeast growth compared to controls (in the absence of compound).

### Toxicity evaluation

#### Cellular toxicity

An MTT kit (Ozbiosciences MT 01000) was used to evaluate the toxicity of hydrazone derivatives. Briefly, the HEK-293 cells were centrifuged for 3 min at 600 rpm and then washed in their medium. 10^5^ cells were placed in 96-well plates in a final volume of 200 μL (plates treated for cell adhesion) and incubated overnight at 37 °C and 5% CO_2_. The medium was then removed and a new medium was added in the presence of the test molecules. The plate was incubated at 37 °C and 5% CO_2_ for 24 h. The cells were washed with PBS and 100 μL of “MTT working solution” was added. After incubation for 4 h at 37 °C and 5% CO_2_, 100 µL of “solubilisation solution” was added to each well. The crystals were dissolved and the plates were read with a spectrophotometer (FLUOstar Omega, BMG LABTECH) at 570 nm with a reference at 650 nm.

#### In vivo toxicity in *Galleria mellonella*

The *G. mellonella* larvae were obtained from the Decathlon campus (Villeneuve d’Ascq, France). No ethical approval statement was required to study the *in vivo* toxicity of our compounds in *Galleria mellonella*. The larvae were kept in food (A mixture of 60 g oatmeal, 20 g honey, 20 g glycerol, 20 g pollen, and 40 g wheat flour) in the dark at 30 °C. Larvae weighing 0.30 ± 0.05 g were used for the toxicity test. On D = 0 the larvae were disinfected using 70% ethanol and 10 µL of the different samples (**PP50** 10 mg/kg, **PP50** 50 mg/kg, and PBS for control) were then injected using an insulin syringe (Terumo Myjector U-100 Insulin) in the penultimate left proto-leg. The viability of the larvae was studied over 7 d.

### Filamentation

A culture of *C. albicans* SC5314 was started the day before the experiments in a liquid Sabouraud medium. After centrifugation at 600 rpm for 3 min, the yeasts were washed twice with PBS medium and then placed in RPMI medium containing 10% foetal calf serum. A total of 10^5^ cells were placed in a 12-well plate, with a final volume of 2 mL of medium, in the presence or absence of **PP50** at different concentrations (2 µg/mL, 4 µg/mL, 8 µg/mL, 16 µg/mL or 32 µg/mL). The plate was incubated at 37 °C during microscopic observation using a Leica DMI8 microscope for 10 h.

### Biofilm assays

#### Impact of PP50 treatment on biofilm formation

A culture of *C. albicans* SC5314 was started the day before the experiments in a liquid Sabouraud medium. After centrifugation at 600 rpm for 3 min, the culture was washed with PBS and then resuspended in RPMI medium. A total of 10^5^ yeasts were placed in a 96-well plate in the presence of the test molecules in RPMI medium (QSF 200 µL). The plate was incubated for 24 h at 37 °C and then washed twice with PBS. The plate was dried for 1 h at 60 °C. Once dry, 200 µL of 0.5% crystal violet was added for 5 min. The plate was washed twice with MQ water and then dried for 1 h at 60 °C. Once dry, the crystal violet was dissolved by adding 250 µL of acetic acid for 20 min. 100 µL of the different conditions were transferred to another plate and then diluted 1/3 before being read with a spectrophotometer (FLUOstar Omega, BMG LABTECH) at 595 nm.

#### Impact of PP50 treatment on early biofilms

A culture of *C. albicans* SC5314 was started the day before the experiments in the liquid Sabouraud medium. The culture was washed with PBS and then resuspended in RPMI medium. A total of 10^5^ yeasts were placed in 96-well plates in RPMI medium (QSF 200 µL). The plate was incubated for 24 h at 37 °C and then washed twice with PBS. 200 µL of RPMI medium containing the test molecules was then added. The plate was incubated for 24 h at 37 °C, washed several times with PBS and dried for 1 h at 60 °C. Once dry, 200 µL of 0.5% crystal violet was added for 5 min. The plate was washed twice with MilliQ water and then dried again for 1 h at 60 °C. Once dry, the crystal violet was dissolved by adding 250 µL of acetic acid for 20 min. 100 µL of the different conditions were then transferred to another plate and then diluted 1/3 before being read with a spectrophotometer (FLUOstar Omega, BMG LABTECH) at 595 nm.

## Results and discussion

### Computational de novo design study

In order to identify putative leads, an *in silico* structure-based drug design approach was considered. The computational study was carried out in two steps. Since TPS2 structural data are available (PDB ID 5DXI, 2 Å), virtual screening using a fragment subset of the ZINC database combined with molecular docking was carried out to identify key fragments as putative leads.

In parallel, an *in situ de novo* design was also performed using LigBuilder 3.0^34^, targeting the C-terminal domain of TPS2 described by Miao et al.^31^ The *de novo* design enabled us to confirm the binding site of trehalose on the surface of the TPS2 protein and to identify several fragments that anchor to the detected cavity (I33, H87, H139) with satisfactory results. Subsequently, these fragments were used for molecular docking and a docking score was calculated.

**Figure 2.**
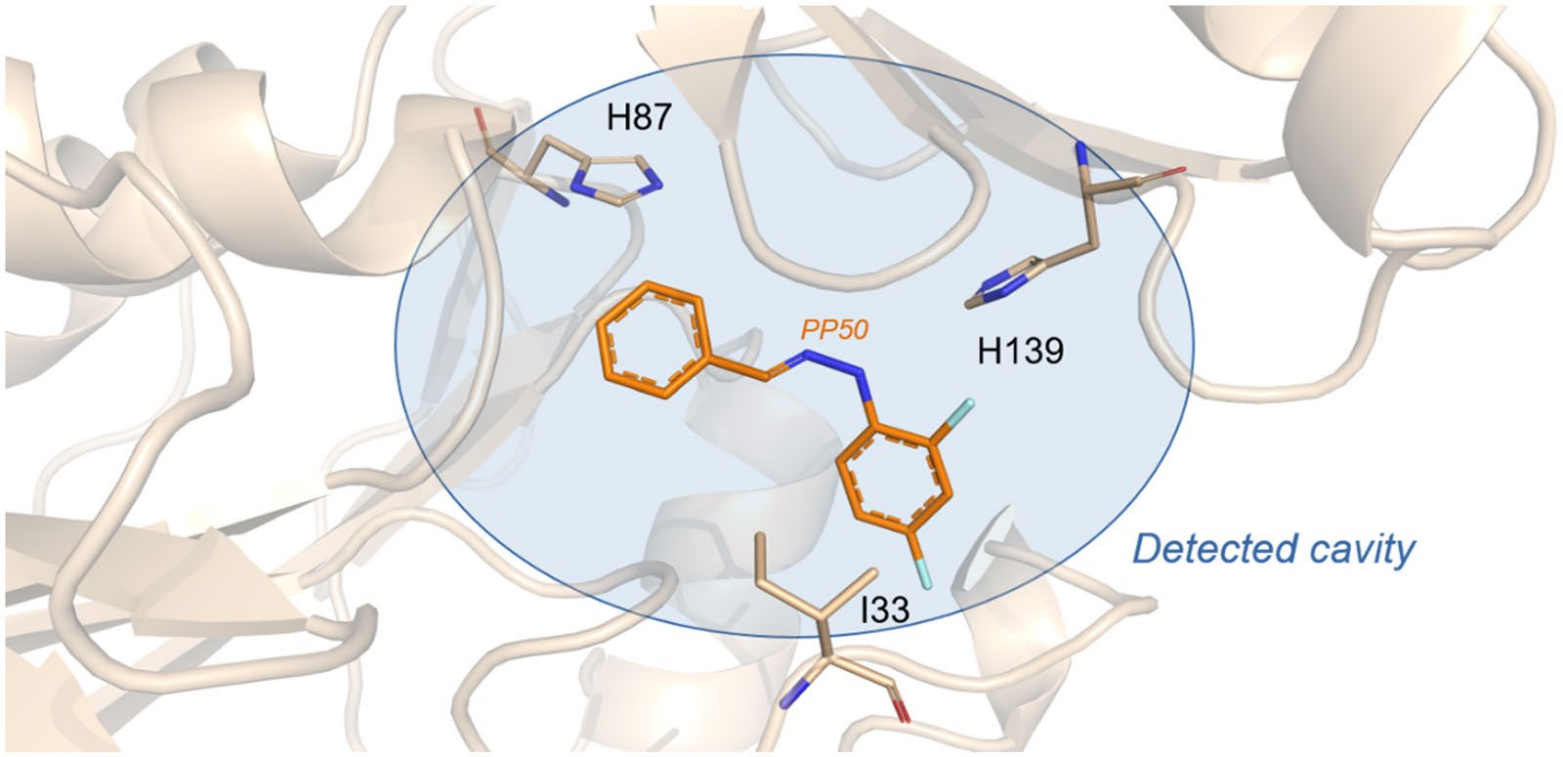
Potent scaffold used as a hit. PP50 is represented as orange sticks with the *E*-hydrazone in blue while TPS2 is in light brown.

Both computational approaches and the evaluation of synthesis accessibility allowed the selection of the hydrazone moiety (Figure 2) as a potential TPS2-inhibitor and thus as a potential antifungal drug.

### Chemistry

**Figure 3.**
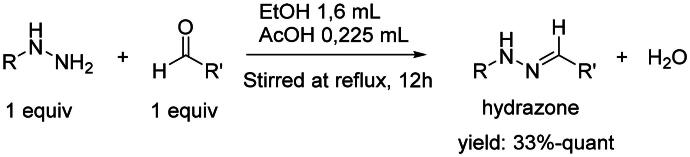
General synthesis pathway of hydrazone derivatives.

The organic synthesis (Figure 3) of hydrazone derivatives is based on the coupling between hydrazine derivatives and benzaldehyde derivatives in ethanol and acetic acid at reflux over 12 h. Purification of the hydrazones was performed by recrystallisation in petroleum ether. The purity of the derivatives was controlled by NMR analysis (^1^H, ^13^C, 2D) and elementary analysis.

In order to study the impact of the substitution (Table 2), different commercial hydrazines (TCI chemical) with (hetero)aromatic or aliphatic substitutions and two benzaldehydes with or without a nitro group at the ortho position were selected.

**Table 2.** Hydrazone substitutions at R and R′ positions.

Compound	R =	R′ =	yield	Compound	R =	R′ =	yield
**PP02**	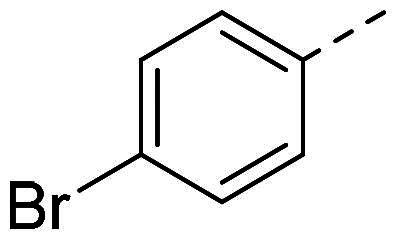	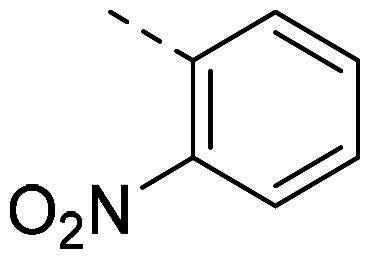	quant	**PP17**	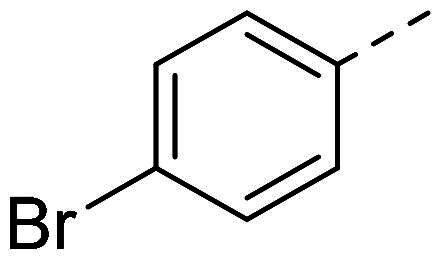	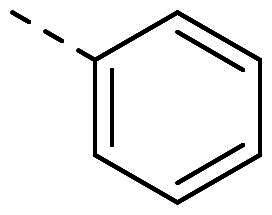	82
**PP04**	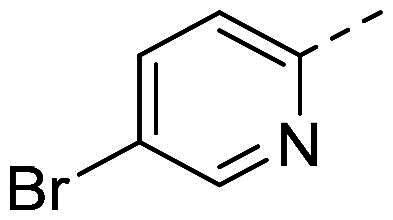	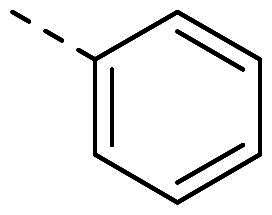	96	**PP25**	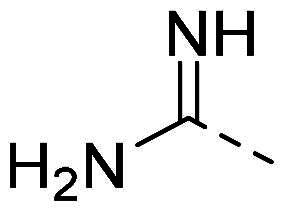	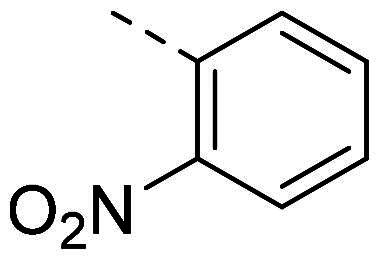	40
**PP11**	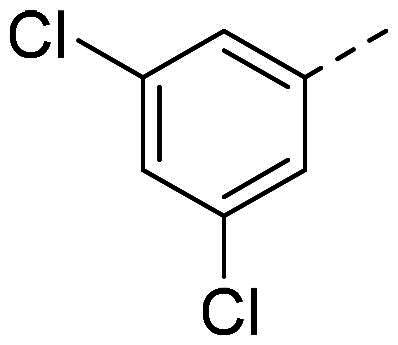	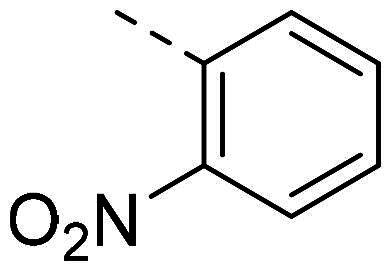	69	**PP28**	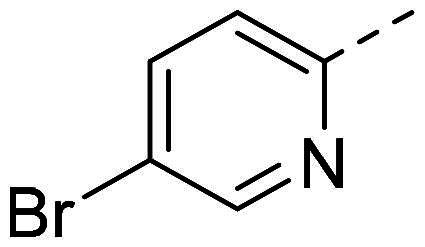	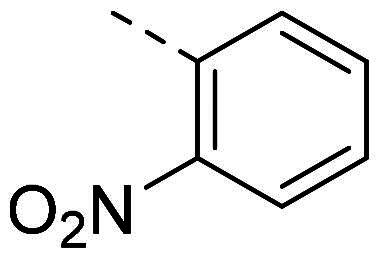	81
**PP12**	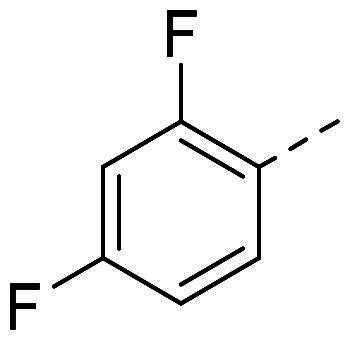	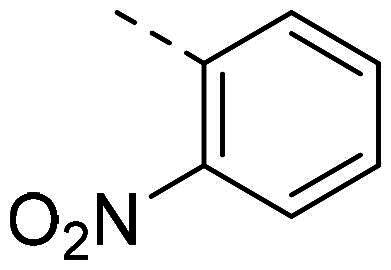	40	**PP29**	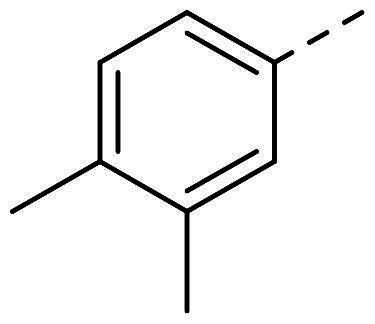	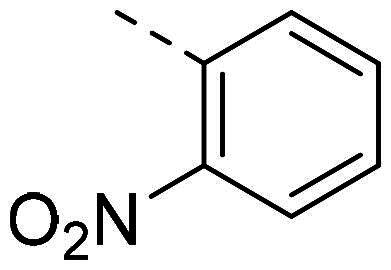	34
**PP13**	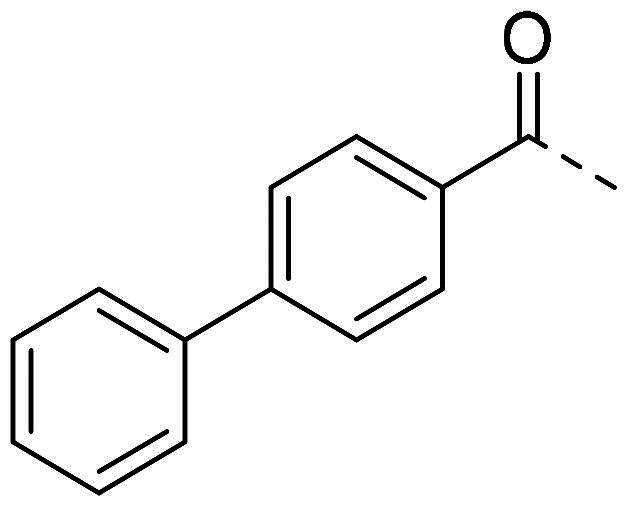	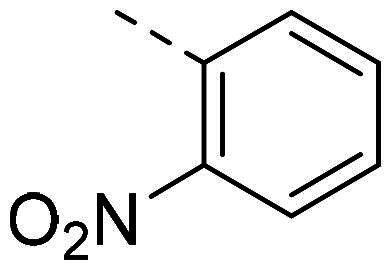	33	**PP49**	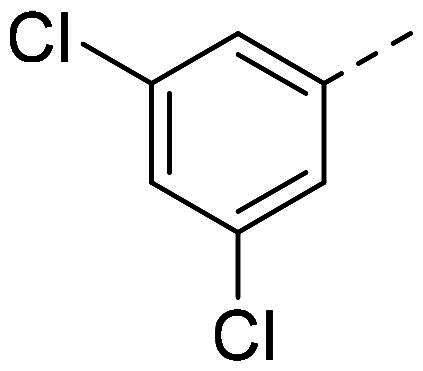	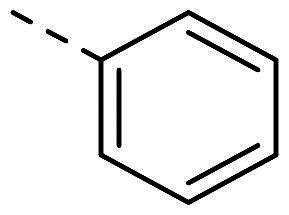	71
**PP14**	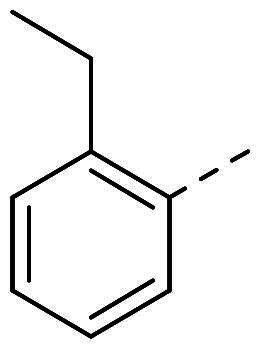	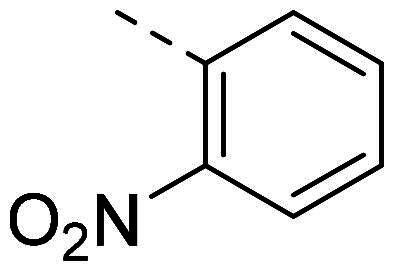	45	**PP50**	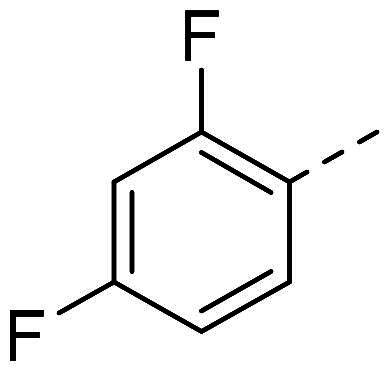	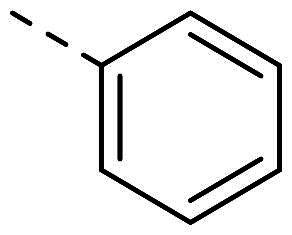	91
**PP16**	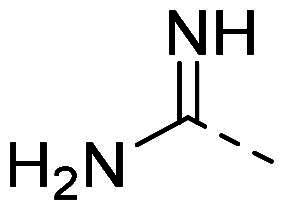	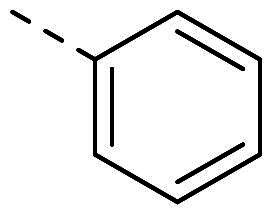	45	**PP53**	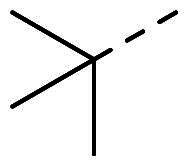	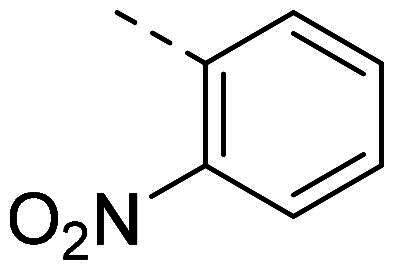	82

**Figure 4.**
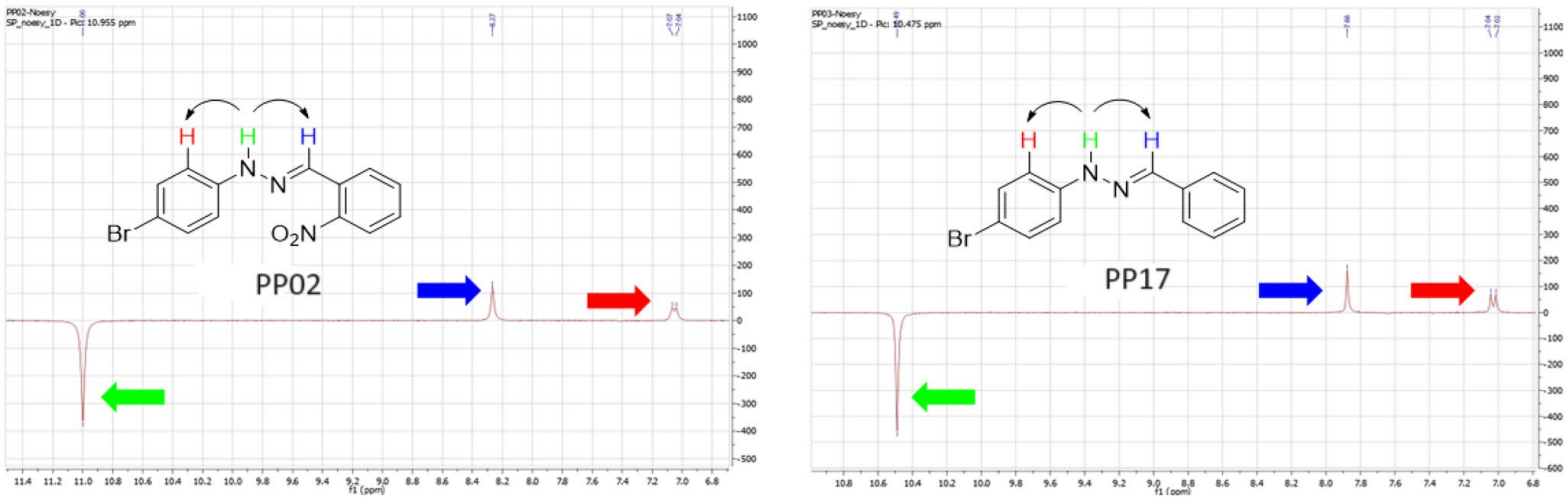
NOESY experiments of PP02 and PP17.

Nuclear Overhauser Enhancement SpectroscopY (NOESY) experiments (Figure 4) were also performed to control the configuration of the compounds. All of the hydrazone derivatives formed had a carbon-nitrogen double bond in their structures; however, two synthetic isomers are then possible in each case, either the “*Z*” configuration or the “*E*” configuration. Only one product was observable by visualisation of the ^1^H NMR spectra, suggesting that the racemic mixture was not obtained, but it was necessary to determine the nature of the obtained configuration. After performing full attribution of ^1^H and ^13^C NMR signals, non-covalent interactions were studied by NOESY experiments. These experiments were carried out for two representative derivatives (Figure 4), in DMSO-*d_6_* at the temperature of 294 K at 300 MHz, for a hydrazone derivative having a nitro group (**PP02**) and the equivalent compound without a nitro group (**PP17**). The correlation of labile hydrogen (in green) with hydrogen located on the carbon involved in the hydrazone double bond (in blue) confirmed in both cases that the “*E*” configuration had been obtained, as for all hydrazone derivatives synthesized from 2-nitrobenzaldehyde or benzaldehyde.

### Biological evaluations

#### Primary screening

The primary biological evaluation consisted of the identification of compounds able to inhibit the growth of *Candida* spp. at a concentration of 32 µg/mL, corresponding to the threshold value beyond which a molecule is not considered as a potential antifungal. This value is relative to the breakpoint value of triazole-resistant strains^42^. Primary antifungal screening was carried out at a single concentration of 32 µg/mL in triplicate, in order to identify interesting hydrazone inhibitors. Primary screening was performed by the successive dilution method described by the Clinical Laboratory Standards Institute with a colorimetric assay based on the conversion of resazurin to resorufin (Alamarblue^TM^ assay, Biorad) by the metabolism of living cells. Fluconazole was used as the positive reference and dimethyl sulfoxide (DMSO) as a negative control in the assay. Samples were prepared in a mixture of DMSO and water, in order to obtain a final concentration of the drug of 32 µg/mL, keeping the final DMSO concentration to a maximum of 1%.

Primary screening (Table 3) was carried out against 10 different strains of *Candida* (Table 1): *C. albicans* SC5314, *C. albicans* clinical strain, *C. albicans* R-echinocandins, *C. auris*, *C. auris* clinical strain, *C. dubliniensis*, *C. tropicalis*, *C. parapsilosis*, C*. glabrata* R-azoles and *C. glabratra* R-echinocandins. Each evaluation was performed on a fresh culture (less than 24 h old) at 37 °C. Strains were cultured on Sabouraud dextrose agar (yeast extract 0.5%, tryptone 1%, NaCl 1%, agar 1.2% and amikacine). One colony was suspended in fresh liquid Sabouraud medium. After one night, the yeasts were centrifuged for 3 min at 600 g and then washed with 1x PBS; a 0.5 McFarland solution was then made from this culture. Twenty µL of the 0.5 McFarland culture were inoculated into 11 mL of RPMI medium (Corning 10–041 CV) and 100 µL of this mixture was placed into the wells of a 96-well plate. 10 µL of the molecule to be tested was then added at the desired concentration followed by 10 µL of Alamarblue^TM^ (Biorad). The plates were incubated at 37 °C for 24 h. The results were read directly by colorimetric observation; yeasts were considered dead when the colour of the culture media in the wells stayed blue.

**Table 3.** Primary screening evaluation and MIC values µg/mL for hydrazone derivatives.

Compound	*C. albicans*	*C. albicans*	*C. albicans*	*C. glabrata*	*C. glabrata*	*C. dubliniensis*	*C. tropicalis*	*C. parapsilosis*	*C. auris*	*C. auris*
	SC5314	17287C305	19505C3469	ATCC 2011	16374C3785	22114C2813	17211M323	14482C4760	1.4.4.10.69	1.4.4.12.29
**PP02**	>32	>32	>32	>32	>32	>32	>32	>32	>32	>32
**PP04**	>32	>32	>32	>32	>32	>32	>32	>32	>32	>32
**PP11**	>32	>32	>32	>32	>32	>32	>32	>32	>32	>32
**PP12**	>32	>32	>32	>32	>32	>32	>32	>32	>32	>32
**PP13**	>32	>32	>32	>32	>32	>32	>32	>32	>32	>32
**PP14**	>32	>32	>32	>32	>32	>32	>32	>32	>32	>32
**PP16**	>32	>32	>32	>32	>32	>32	>32	>32	>32	>32
**PP17**	32	32	32	32	32	>32	>32	16	>32	>32
**PP25**	>32	>32	>32	>32	>32	>32	>32	>32	>32	>32
**PP28**	>32	>32	>32	>32	>32	>32	>32	>32	>32	>32
**PP29**	>32	>32	>32	>32	>32	>32	>32	>32	>32	>32
**PP49**	>32	32	32	>32	32	32	>32	8	32	32
**PP50**	32	32	>32	32	32	32	>32	16	>32	>32
**PP53**	>32	>32	>32	32	32	>32	>32	16	>32	>32
**Fluconazole**	0,25	0,5	>256	16	256	025	64	2	>256	0,5
**Caspofungin**	0,06	4	<0,008	0,015	>8	0,12	0,25	0,5	1	0,25

Primary screening of the 14 hydrazone analogues showed that the derivatives with the nitro group did not have any antifungal activity, except for **PP53** which exhibited an antifungal effect on *C. parapsilosis*. Conversely, four hydrazones without a NO_2_ substitution demonstrated notable antifungal activity at 32 µg/mL.

#### Minimum inhibitory concentration (MIC) determination

The MIC values (Table 3) were evaluated against the different strains of *Candida* for the compound which demonstrated antifungal activity during the preliminary screening assay, at 32 µg/mL (In Table 3, >32 µg/mL means that the MIC was not determined because the compound was not active at 32 µg/mL during the preliminary screening). The same colorimetric assay was used as for the primary screening, for eight different concentrations (from 0.25 to 32 µg/mL) of the compounds, in triplicate. Interestingly, the best antifungal activities were observed against *C. auris* and *C. parapsilosis* for **PP49** and *C. parapsilosis* for **PP50** and **PP53**. **PP17** demonstrated antifungal activity against different stains of *Candida* with MIC values between 8 and 32 µg/mL. Moreover, among the hydrazone derivatives, **PP17** and **PP50** were the only compounds which demonstrated an antifungal effect on the reference strain of *C. albicans* SC5314, which is the strain used in the biofilm model in our laboratory.

#### *In vitro* toxicity

**Figure 5.**
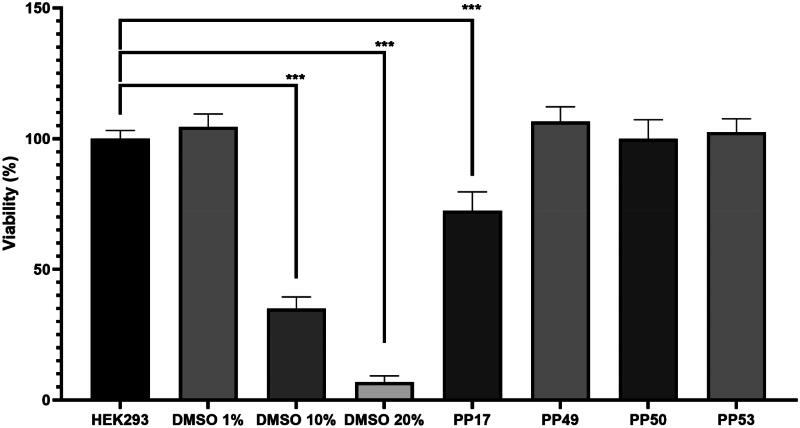
Viability (%) of Human Embryonic Kidney 293 cells (HEK293) without treatment or with 1% DMSO, 10% DMSO, 20% DMSO, PP17 (32 µg/mL), PP49 (32 µg/mL), PP50 (32 µg/mL), PP51 (32 µg/mL) and PP53 (32 µg/mL).

Antifungal drugs are usually administered to patients with comorbidities, so it is essential to evaluate the toxicity of the compounds to anticipate any potential side-effects. Toxicity (Figure 5) was determined on Human Embryonic Kidney 293 cells (HEK293). The toxicity assay is based on a viability test of HEK293 cells exposed to the drugs. The assay is a colorimetric test based on the conversion of tetrazolium salts (MTT) to formazan crystals by the respiratory chain of the cells. Toxicity was determined for the active hydrazones and for an unique dose of 32 µg/mL, in triplicate. Among the tested hydrazones, only one compound was toxic to human cells, **PP17** at 32 µg/mL. Compound **PP50** was retained for further experiments.

#### *In vivo* toxicity

Since the welfare of animals is guaranteed by the 3Rs rules (Replacement, Reduction and Refinement), the researchers are incited to replace traditional mammalian models, especially the murine model. In the last few decades, the insect larva of *G. mellonella* is described as an *in vivo* model to determine the toxicity of chemicals compounds or to evaluate new drugs in pathogenic context^43^^,^^44^. This model presents many advantages as: immune response similar to those of mammals, rapid, low cost, easy to use, no ethical constraints^45^. Moreover, several studies demonstrate that the response of *G. mellonella* larvae to toxically studies is strongly correlated with the results obtained with *in vitro* cell lines models and murine models^45^. For these reasons, we decided to use *G. mellonella* in order to study the *in vitro* toxicity of **PP50.**

Larvae of the sixth developmental stage of *Galleria mellonella* were obtained from the Decathlon campus (Villeneuve d’Ascq, France). Larvae were stored in food (a mixture of oatmeal, honey, glycerol, pollen, and wheat flour) in the dark at 30 °C. Larvae that weighed 0.30 ± 0.05 g were used within 1 week of receipt. For each treatment, 12 healthy larvae were placed in sterile 9-cm Petri dishes lined with Whatman filter paper.

**PP50** was dissolved in DMSO and this solution was diluted in mQ H_2_O to the required concentrations. The toxicity of **PP50** was evaluated at 10 mg/kg and 50 mg/kg. Twelve larvae were used for each condition. As controls, equal numbers of larvae kept in a second Petri dish were injected with a mixture of DMSO/PBS (10/90). Samples (10 μL) were injected into the *G. mellonella* haemocoel through the penultimate right pro-leg using an insulin syringe (Terumo Myjector U-100 Insulin). The inoculated larvae were incubated at 30 °C for 7 days. Larvae were individually examined for pigmentation (melanisation is correlated with death in larvae) and the number of live larvae was recorded every day. Larvae that did not show any movement in response to touch were considered dead.

**Figure 6.**
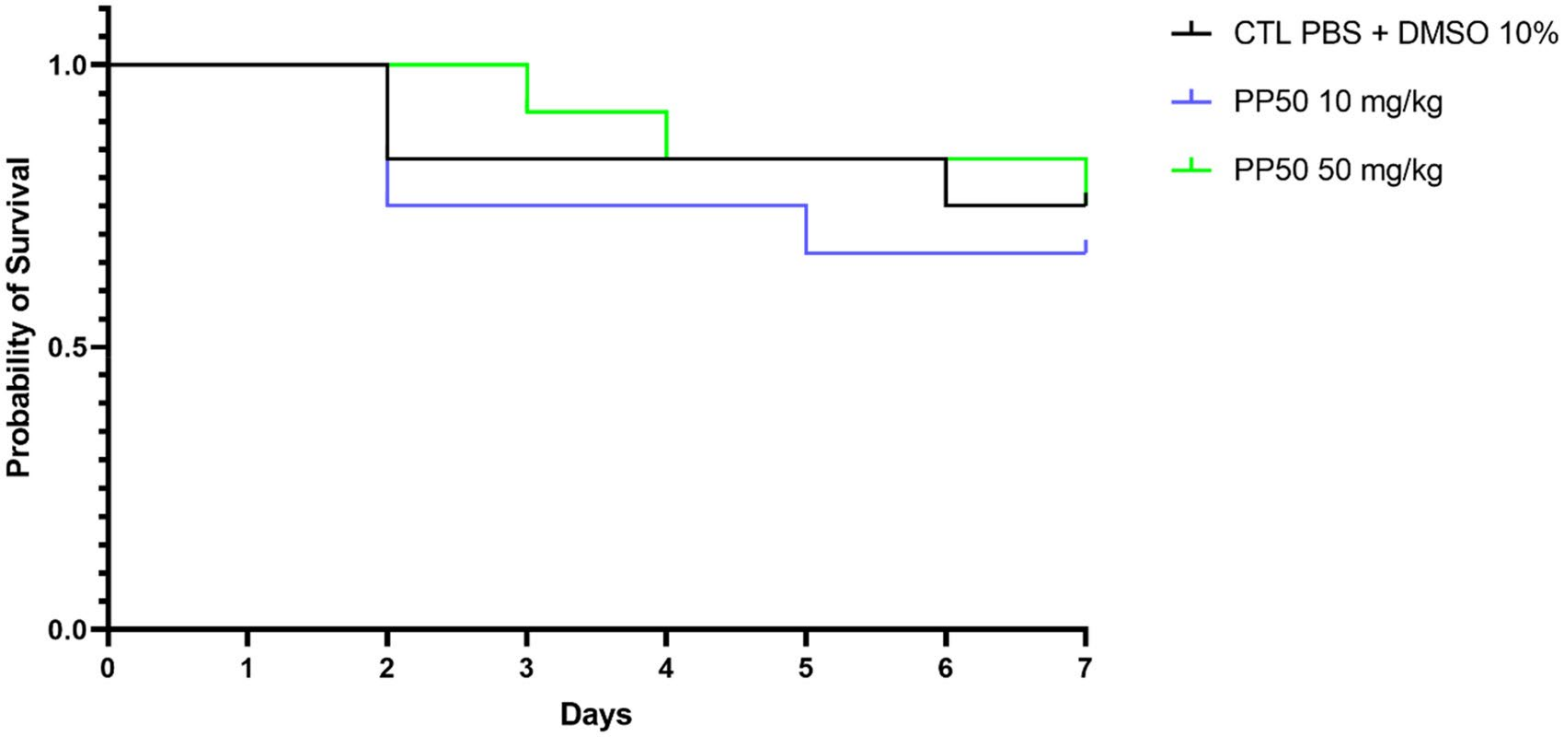
Probability of survival of *Galleria mellonella* with control injection (PBS + 10% DMSO) or treatment (PP50 at doses of 10 mg/kg or 50 mg/kg).

No toxicity (Figure 6) was observed in *G. mellonella*, even with the higher concentration of **PP50** (50 mg/kg).

The *in vitro* and *in vivo* toxicity evaluations led us to consider hydrazone as a promising chemical family to develop antifungal drugs.

#### Biofilm and filamentation assays

**Figure 7.**
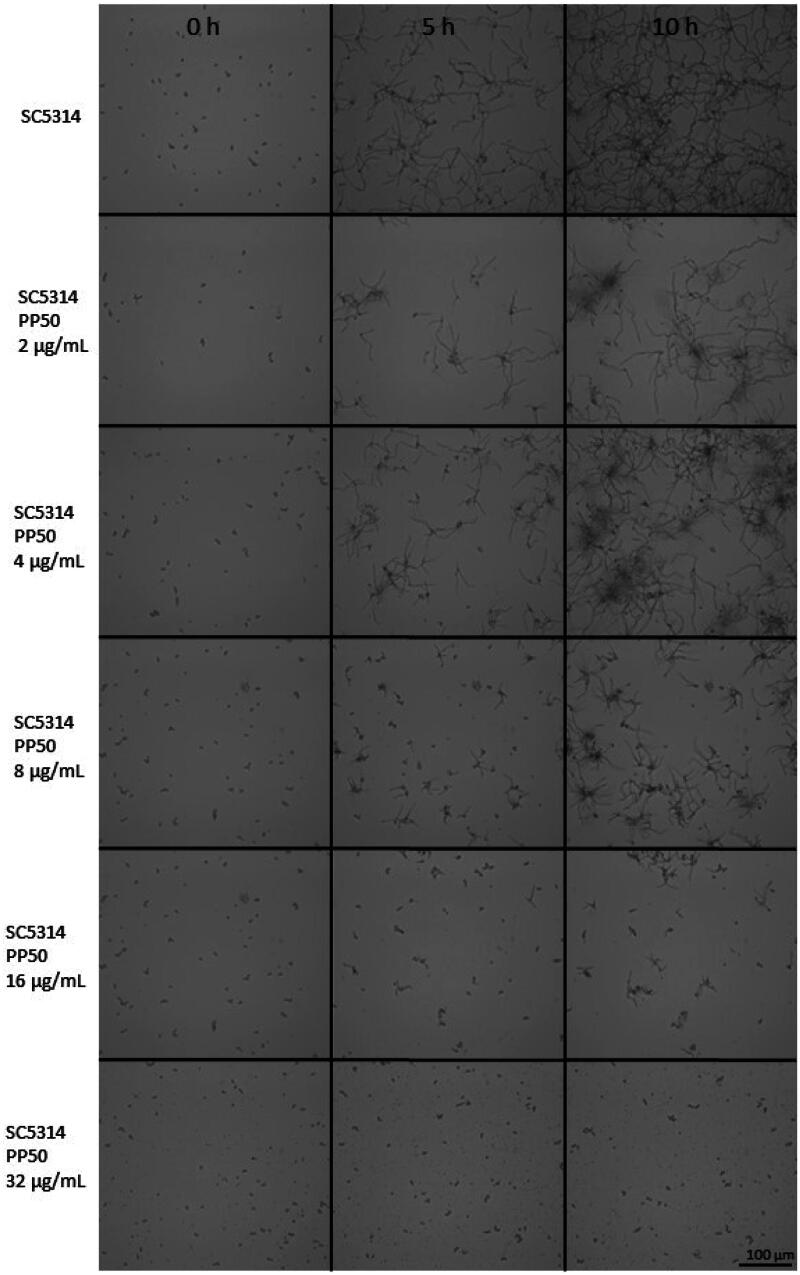
Filamentation of *C. albicans* (SC5314 strain) in the presence of PP50 at 0 µg/mL (control), 4 µg/mL, 8 µg/mL, 16 µg/mL and 32 µg/mL, for 0 h, 5 h and 10 h.

The formation of *Candida* biofilms occurs in different developmental phases: initial adhesion and colonisation, cell division, proliferation then maturation. The complex structure of the mature biofilm allows the yeast to take up nutrients and resist antifungal drugs. Moreover, since it has been reported that 50% of cases of systemic candidiasis in adult patients and 30% of younger population deaths are correlated with the presence of biofilms during infection, it appears essential to study the impact of new molecules on biofilm formation and the filamentation process. Filamentation plays a critical role in the development of mature and highly structured *C. albicans* biofilms^46^. It was previously demonstrated that yeasts that were not able to switch to the hyphal form cannot form biofilms^47^. In this context, it was decided to evaluate the impact of **PP50** (Figure 7) on filamentation by *C. albicans* SC5314 strain at 37 °C for different times: 0 h, 5 h, and 10 h, and at different concentrations of **PP50**: 0 µg/mL (control), 4 µg/mL, 8 µg/mL, 16 µg/mL and 32 µg/mL.

At 5 h (Figure 7), *C. albicans* (SC5314 strain) developed hyphal forms while at 10 h it started to form early biofilms. Filaments obtained with **PP50** (4 µg/mL) were smaller after 5 h. Moreover, **PP50** led to sparse and less structured early biofilms compared to untreated culture. Interestingly, with **PP50** (16 µg/mL), *C. albicans* did not convert to the hyphal form and was not able to form biofilms. This study shows that **PP50** had a strong effect on filamentation and on early biofilm formation by *C. albicans*.

**Figure 8.**
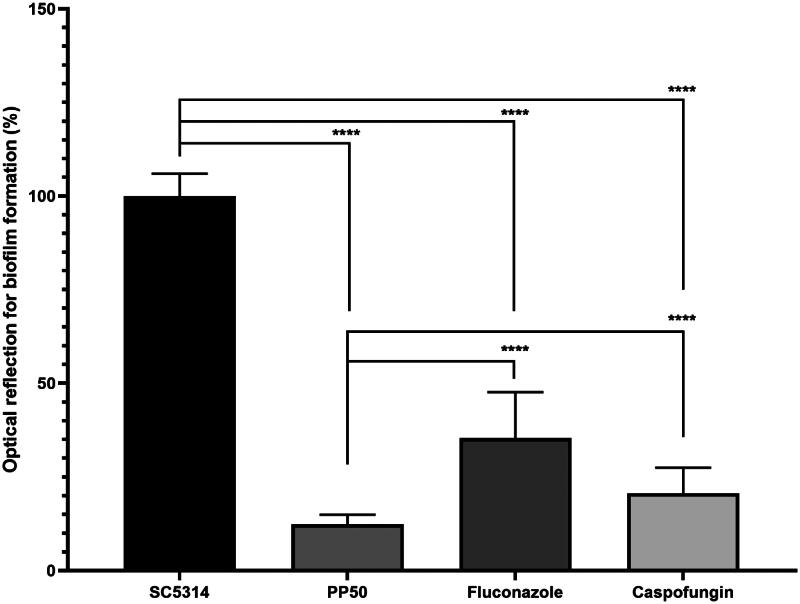
Optical reflection of biofilm formation (%) of *C. albicans* (SC5314 strain) in the absence of drug or in the presence of PP50 (32 µg/mL), fluconazole (32 µg/mL) or caspofungin (32 µg/mL).

We then conducted further studies to investigate the impact of **PP50** on biofilm formation (Figure 8). Briefly, *C. albicans* were cultured in 96-well plates with **PP50**, fluconazole or caspofungin at a concentration of 32 µg/mL. After 24 h at 37 °C, biofilm formation was studied using crystal violet assays.

Biofilm formation was altered with all compounds. Fluconazole inhibited biofilm formation by 65%, caspofungin by 80% and **PP50** by 90%. These results (Figure 8) were obtained at 32 µg/mL, corresponding to the MIC value of **PP50**, 533x MIC value of caspofungin and 128x MIC value of fluconazole. These results suggested that **PP50** is more effective than fluconazole and caspofungin at inhibiting biofilm formation by *C. albicans* (SC5314 strain).

**Figure 9.**
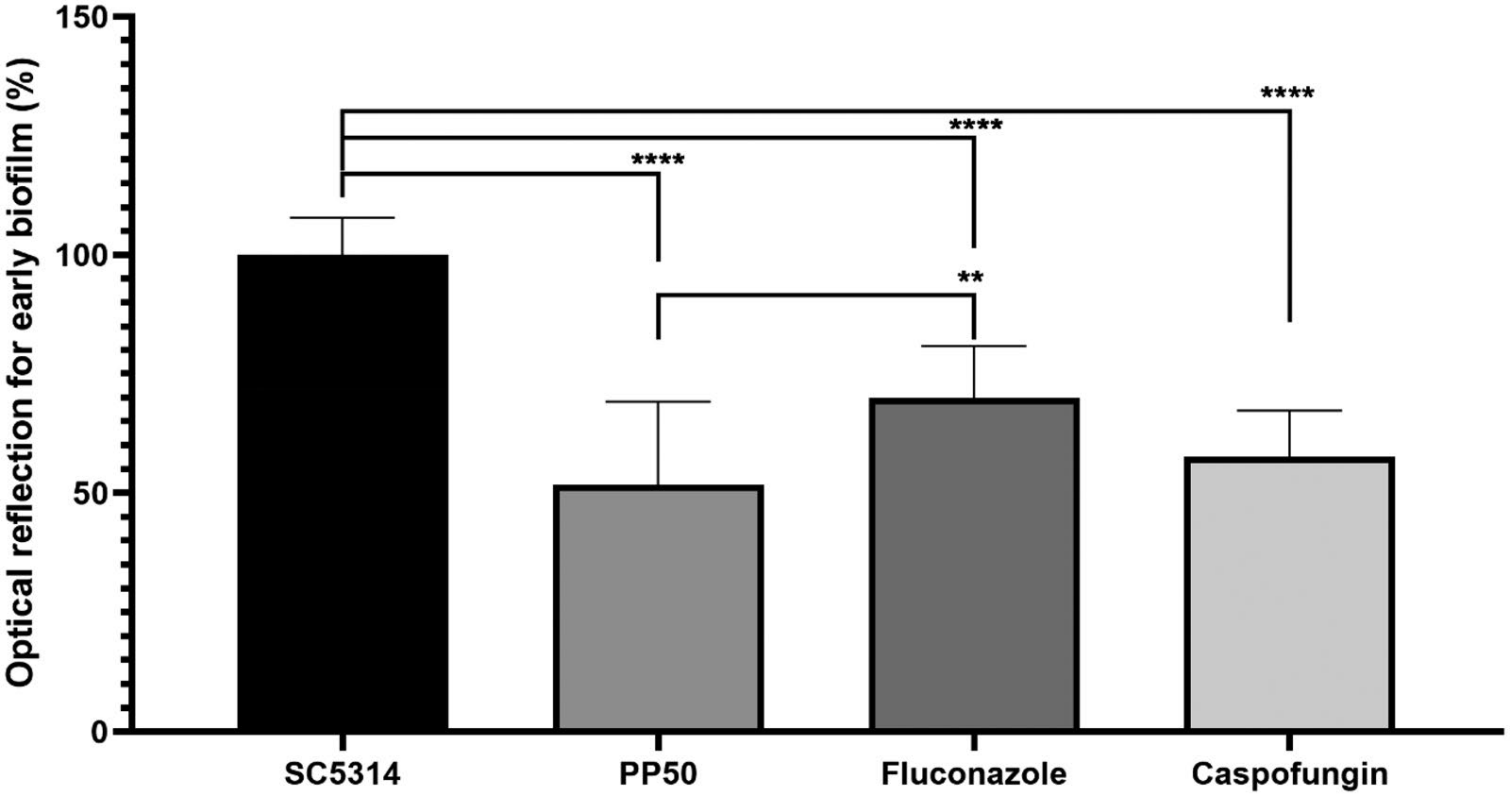
Optical reflection (%) of early biofilms of *C. albicans* (SC5314 strain) in the absence of drug or in the presence of PP50 (32 µg/mL), fluconazole (32 µg/mL) or caspofungin (32 µg/mL).

The impact of **PP50** treatment on early biofilm formation was then investigated (Figure 9). Briefly, *C. albicans* were cultured in 96-well plates with **PP50**, fluconazole or caspofungin at a concentration of 32 µg/mL. After 24 h at 37 °C, biofilm formation was studied using crystal violet assays.

At 32 µg/mL (Figure 9) **PP50** resulted in strong inhibition of early biofilm formation (50%) compared to fluconazole (30%) and caspofungin (40%). Nevertheless, it is important to note that the experiments were carried out at a dose of 32 µg/mL, which corresponds to the MIC value of **PP50**, 128x the MIC of fluconazole and 533x the MIC of caspofungin. These results suggest that **PP50** is more effective at destroying early biofilms than fluconazole or caspofungin.

## Conclusion

Since TPS2 was identified as an essential pathway to stress resistance, virulence and prevention of hyphal development in *C. albicans*, a drug design strategy based on a *de novo design* computational study was undertaken in order to identify new inhibitors of TPS2. From the 3-dimensional structural data of TPS2, hydrazone derivatives were identified as potential antifungal drugs. The synthesis and biological evaluation of hydrazone derivatives suggest that this drug family presents moderate antifungal activity and no toxicity, and also has a significant impact on the filamentation process, on biofilm formation and on early biofilms. This result is very interesting since filamentation and biofilms are very important for the virulence and infectivity of *C. albicans*. Hydrazone seems to be an interesting scaffold to develop antifungal drugs which could prevent the development of invasive candidiasis, particularly in fragile patients.

These results suggest that **PP50** is a good candidate to attenuate virulence of *Candida.* Further experiments will be performed in a murine model to evaluate the antifungal effect of this drug in an invasive candidiasis context.

## Supplementary Material

SuppFASTA.pdf

## Data Availability

The datasets presented in the current study are available from the corresponding author upon reasonable request.
