# Supplementary material for: Antifungal and anti-biofilm effects of hydrazone derivatives on Candida spp
Source: J Enzyme Inhib Med Chem. 2024 Nov 26;39(1):2429109. doi: 10.1080/14756366.2024.2429109 (PMC11600518; doi:10.1080/14756366.2024.2429109)
Supplement: SuppFASTA.pdf [file IENZ_A_2429109_SM7273.pdf]

>5DXI_1|Chains A, B|trehalose-6-phosphate phosphatase|Candida albicans (237561)
SNAYTPALNRPLLLNNYKESQRRLFLFDYDGTLTPIVQDPAAAIPSDKLNRILDVLSSDPKNQIWIISGRDQA
FLEKWMGNKNVGLSAEHGCFMKDIGSKEWVNLAASFDMSWQEKVDDIFKYYTEKTPGSNIERKKVALTWHYRR
ADPDLGNFQAEKCMKELNDTVAKEYDVEVMAGKANIEVRPKFVNKGEIVKRLVLHPHGAKQEKHPTGHCTKDI
PIEELPDFMLCLGDDLTDEDMFNSLNEINKKWKGDNRPTNKFGSYGVYPVAVGPASKKTVAIAHLNEPRQVLE
TLGLLAGLVS
